# Contribution of rare mutational outcomes to broadly neutralizing antibodies: Rare features of unique anti-viral antibodies

**DOI:** 10.3724/abbs.2022065

**Published:** 2022-06-09

**Authors:** Anqi Feng, Qian Hao, Leng-Siew Yeap

**Affiliations:** Shanghai Institute of Immunology State Key Laboratory of Oncogenes and Related Genes Department of Immunology and Microbiology Shanghai Jiao Tong University School of Medicine Shanghai 200025 China

**Keywords:** somatic hypermutation, AID, broadly neutralizing antibodies, indels, long CDR H3

## Abstract

Antibodies are important immune molecules that are elicited by B cells to protect our bodies during viral infections or vaccinations. In humans, the antibody repertoire is diversified by programmed DNA lesion processes to ensure specific and high affinity binding to various antigens. Broadly neutralizing antibodies (bnAbs) are antibodies that have strong neutralizing activities against different variants of a virus. bnAbs such as anti-HIV bnAbs often have special characteristics including insertions and deletions, long complementarity determining region 3 (CDR3), and high frequencies of mutations, often at improbable sites of the variable regions. These unique features are rare mutational outcomes that are acquired during antibody diversification processes. In this review, we will discuss possible mechanisms that generate these rare antibody mutational outcomes. The understanding of the mechanisms that generate these rare mutational outcomes during antibody diversification will have implications in vaccine design strategies to elicit bnAbs.

## Introduction

**Figure 1:**
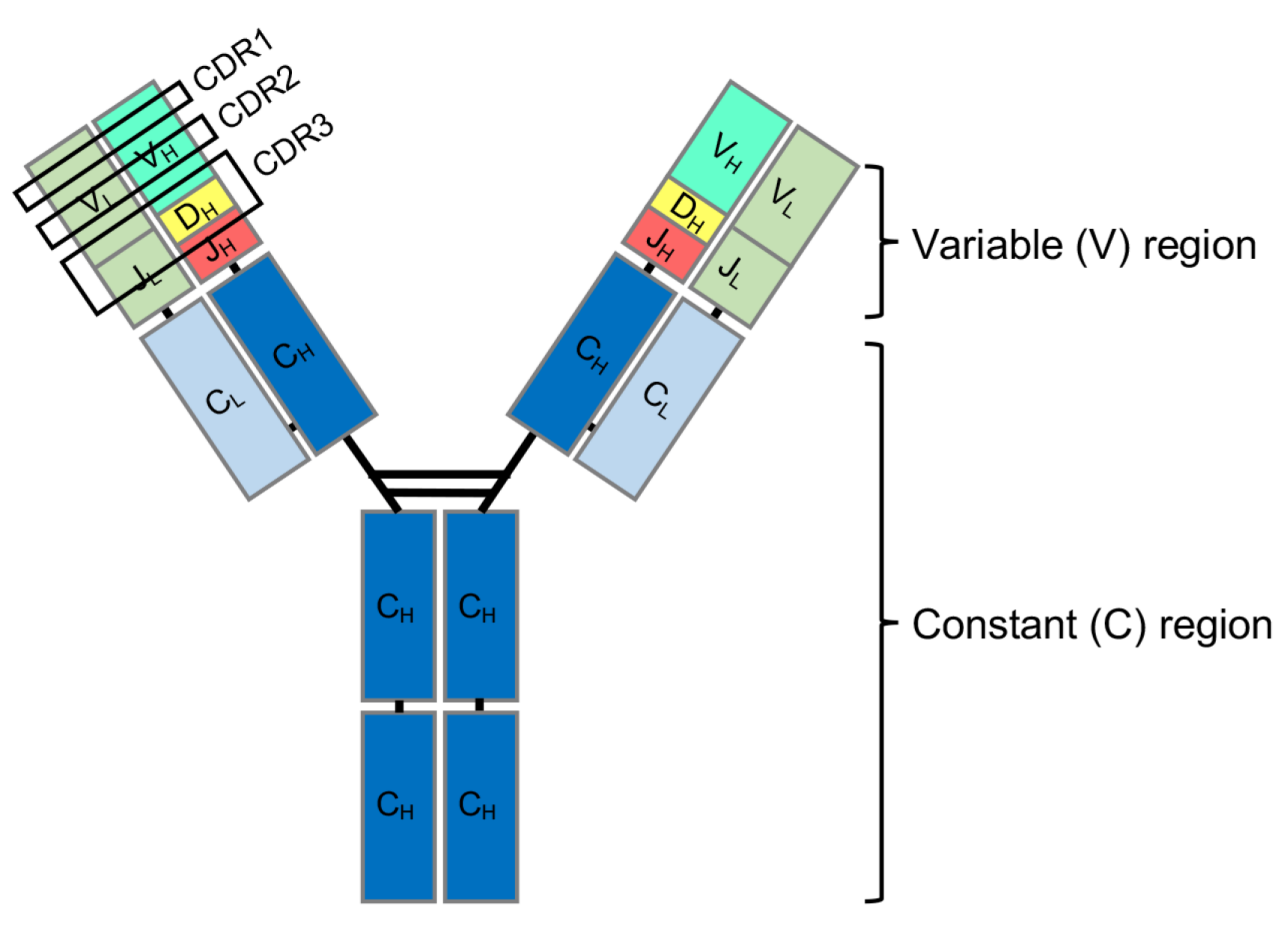
Figure 1 Structure of antibody Antibodies consist of two identical immunoglobulin heavy chains (IgH) and two identical light chains (IgL). The N-terminus regions of the IgH and IgL are the variable (V) regions, which are responsible for antigen binding. Complementarity-determining regions (CDRs) including CDR1, 2, and 3 are indicated as regions in black boxes. The C-terminus regions of the IgH and IgL are the constant (C) regions which are responsible for the effector functions of the antibody.

The diversity of B cell receptor (BCR), or its secreted form, antibody, is a hallmark of adaptive immunity, which allows the recognition and removal of diverse foreign antigens that are harmful to our bodies. The antibody is a y-shaped molecule that is secreted by plasma cells. Antibodies are immunoglobulin (Ig) proteins consisting of four polypeptides: two identical immunoglobulin heavy chains (IgH) and two identical light chains (IgL) [1]. The N-terminus region of the IgL and IgH which is highly variable, hence called the variable (V) region, is responsible for the binding to foreign antigens, while the less variable region at the C-terminus, called the constant (C) region, is responsible for the effector functions of the antibody ( Figure 1). Amongst the variable regions in the heavy (V _H_) and light (V _L_) chains, there are three hypervariable regions, called the complementarity-determining regions (CDR1, 2, and 3) in which the antigen binds. The diversity of the N terminus of antibodies is mainly contributed by two processes involving programmed DNA lesions of the *Ig* genes, V(D)J recombination and somatic hypermutation (SHM) [2].

V(D)J recombination is the first process that generates a diverse primary BCR repertoire and it occurs during the development of B cells in the bone marrow. V(D)J recombination is a process that cuts and joins the numerous variable (V), diversity (D), and joining (J) segments to form V region exons [3]. The V region exon of the IgH consists of the V, D, and J segments of *IgH*, while the V region exon of the *IgL* consists of V and J segments of either *Igκ* or *Igλ*. The expression of RAG endonuclease during the development of B cells initiates the process of V(D)J recombination and the gene segments are joined by DNA repair mechanisms. The CDR3 of *IgH* (CDR H3) is the most diverse amongst the CDRs, as it consists of sequences from the V, D, and J segments, in addition to junctional nucleotides added by terminal deoxynucleotidyltransferase (Tdt) during the recombination process [ 4, 5] . The process of V(D)J recombination occurs independently of antigen stimulation, thus the primary BCR repertoire that is generated by this process has low affinity for antigen binding.

Matured naïve B cells that exit the bone marrow migrate to the periphery where they have a chance to encounter cognate antigens. Upon activation by cognate antigens in the periphery, matured naïve B cells undergo SHM, which introduces point mutations at high frequencies and sometimes insertions and deletions (indels) in the V region exons [6]. This process occurs in germinal centers (GCs), a specialized compartment in the secondary lymphoid organs. B cells that have mutational outcomes that increase binding affinity with the antigens will be selected, while B cells with deleterious mutational outcomes will be negatively selected [7]. Through multiple rounds of SHM, clonal expansion, and selection for high-affinity BCRs, affinity maturation of the antibody response is achieved. GC B cells differentiate into plasma cells which secrete high affinity antibodies for humoral response or memory B cells for long-term immune memory.

SHM is initiated by activation-induced cytidine deaminase (AID) [ 8, 9] . AID also initiates *IgH* class switch recombination (CSR), a DNA deletional recombination process that changes the isotype of the antibody, for example from IgM to IgG, for the effector functions of the antibodies [10]. CSR can occur inside or outside the GC [ 11, 12] . AID is a cytidine deaminase that deaminates cytidines to uridines in single-stranded DNA (ssDNA) [ 13– 16] . AID activities require transcription which provides the ssDNA substrate. At the DNA level, AID preferentially targets specific sequence motifs (“hotspots”) such as the DGYW motif [17]. The repair of AID lesion in an error-prone manner by the base excision repair (BER) and mismatch repair (MMR) pathways leads to different mutational outcomes, which increases the diversity of the variable region exons [ 18, 19] . For example, A:T mutations at the V exons are generated by the MMR pathway through gap filling by error-prone DNA polymerase [19]. The diverse mutational outcomes of AID further generate diversity in the V(D)J exons.

**Figure 2:**
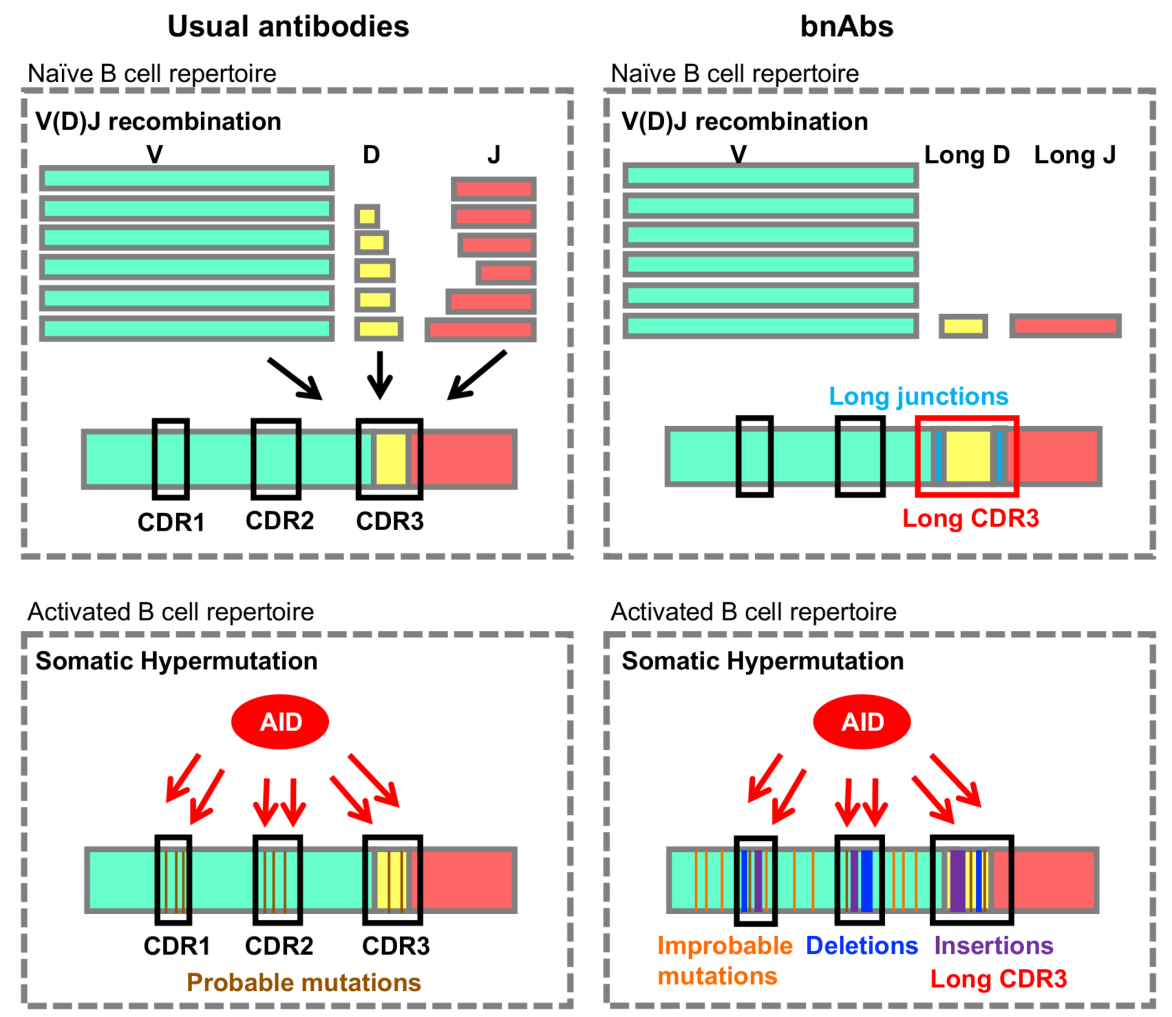
Figure 2 Possible mechanisms that generate rare mutational outcomes in broadly neutralizing antibodies during antibody diversification processes Rare mutational outcomes such as long CDR3 could be generated during V(D)J recombination (top right) or SHM (bottom right). AID activities during SHM could generate rare mutations at improbable nucleotides, deletions, and insertions, which could lengthen the CDR3. bnAbs: broadly neutralizing antibodies.

Although point mutations are the most common outcomes during SHM, rare outcomes such as indels are found to be over-represented in unusual antibodies such as the rare anti-viral broadly neutralizing antibodies (bnAbs) [20]. The discoveries of anti-HIV and anti-influenza bnAbs in recent years have renewed hope for a successful HIV vaccine and a universal vaccine for influenza [ 20, 21] . Anti-HIV bnAbs have unique features such as frequent indels, extensive levels of SHMs including mutations at the framework regions and long CDR H3s [20]. These unusual features are rare outcomes of antibody diversification processes ( Figure 2). In this review, we will discuss the potential mechanisms underlying these rare mutational outcomes and implications for vaccine strategies to elicit anti-viral bnAbs.

## Insertions and Deletions Are Rare Products of SHM

Indels are byproducts of SHM process [22]. They were found in the V(D)J exons of human tonsillar GC B cells in the late 1990s [ 23, 24] . Goosens *et al*. [23] found that indels accounted for ~6% of somatic mutations introduced into the V(D)J exon of human tonsillar GC B cells and over 40% of out-of-frame rearrangements of GC B cells harbored indels. The indel length ranged from 1-bp insertion to >268-bp deletion, and insertions were mostly duplications of adjacent sequences. According to the study by Wilson *et al*. [24] in 1998, 6 indels were from 110,000 nucleotides sequenced from 395 cDNA clones of human tonsillar IgG memory cells, resulting in a frequency of <2% clones analyzed. The indels were in-frame and short, varying from 1 to 6 amino acids. As indels were not found in naïve B cells, the indels were associated with the process of SHM rather than V(D)J recombination. The mechanisms that generate indels have not gained much attention from researchers likely because indels are low-frequency mutational events. Furthermore, indel events could arise during PCR or sequencing experiments, resulting in difficulties in distinguishing *bona fide* SHM-initiated indel events from artifacts.

### Insertions and deletions in anti-viral broadly neutralizing antibodies

In the 2010s, with the advancement of probes used for detecting antigen-specific memory B cells in fluorescent-activated sorting experiments, a panel of potent bnAbs against human immunodeficiency virus 1 (HIV-1) were isolated from a small population of HIV-infected patients [ 25, 26] . A common feature of these rare bnAbs is the frequent occurrence of indels in the V(D)J exon. In a study by Kepler *et al*. [27], 30 out of the 108 (28%) anti-HIV bnAbs surveyed contain insertions, and 23 out of the 108 (21%) bnAbs contain deletions. These indels are found in the CDR1 and CDR2 regions of both *IgH* and *IgL* variable region exons and are important for the neutralizing activity of the bnAbs. For example, in a longitudinal study of the CH31 clonal lineage antibodies [ 28, 29] , the authors showed that broad HIV neutralization was acquired only after a 6-nt deletion and a 33-nt insertion happened around the CDR H1 [27]. The isolation of these bnAbs has reignited hope for a successful HIV vaccine. In this light, the question of how indels are generated during antibody diversification is of utmost importance.

Indels have also been found to play important roles in neutralizing antibodies against influenza. For example, a 3-aa insertion near the CDR H2 in the 2D1 human monoclonal antibody confers neutralizing activities of the antibody to the 1918 and 2009 HIN1 influenza virus [30]. The insertion changes the structure of the antibody, causing enhancement of antibody binding and function. The example of this anti-influenza antibody and the anti-HIV bnAbs described above show that indels in the V(D)J exon play beneficial roles to certain anti-viral antibodies. Therefore, increasing the probability of B cells carrying indel events during antibody diversification processes could be helpful to select bnAb precursors.

### Large insertions in anti-malaria antibodies

Apart from the discoveries of the relatively short indels in the anti-HIV bnAbs, recent research shows that insertions as long as 98 amino acids could occur in antibody genes, such as those involving the insertion of *LAIR1* sequence in antibodies against malaria variant antigens [ 31, 32] . The insertion between the V and DJ segments allows the antibody to acquire broad reactivity against malaria variant antigens [31]. This kind of large insertions is different from the short insertions in human tonsillar GC B cells, as the large insertions involve sequences from other chromosomes while the short insertions are mostly duplications of the adjacent *Ig* sequence. Thus, the mechanism that generates the large insertions could be different from the mechanism that generates the short insertions.

### Possible mechanisms of indel generation

How indels at the *IgV* regions are generated during *Ig* diversification is not well understood, partly due to the lack of a model system that can robustly assay these rare mutational events. Nonetheless, the exploration of mechanisms of CSR provided some clues to how deletions could be generated [ 18, 19] . During CSR, AID targeting of the *IgH Switch* ( *S*) regions results in the downstream constant region being juxtaposed next to the *IgH V(D)J* exon. The *S* regions are densely packed with AID-preferred DNA motifs which are targeted by AID at high frequency [33]. AID-initiated lesions can be converted to DNA double strand breaks (DSBs), which then activate the DSB response (DSBR) pathway. The long-range joining of two broken *S* regions at the *IgH* locus leads to CSR [10]. Broken ends at a single *S* region that are joined imperfectly by the non-homologous end joining (NHEJ) or alternative end joining (A-EJ) pathways can lead to internal deletions [34]. Thus, deletions at *S* regions involve the generation of DSBs and DNA end joining factors.

In the V region exons, deletions occur frequently in regions that are highly targeted by AID, such as the CDR regions [35]. This finding was obtained from studies in mouse models carrying *passenger-Ig* alleles that do not encode functional Ig proteins [ 35, 36] . While the CDRs are somewhat enriched for AID-preferred DNA motifs, they are not as densely packed as the S regions [33]. It is still unclear whether DSBs in the V regions lead to deletions, as their frequencies were too low to be detected in previous mouse genetic studies [ 18, 19] . In this context, the *passenger-Ig* allele system could be useful to assay deletion frequencies in the DNA repair-deficient mouse models. However, such studies would require complex breeding strategies involving various DNA repair factors and have yet to be reported.

Several distinct mechanisms have been proposed to explain how insertions are generated. Insertions occurring around the repetitive tracts may be explained by the DNA slippage model or the DNA misalignment model [ 24, 37, 38] . Several reports showed that insertions could be generated during Cas9-induced DSB repair [ 39– 42] . DNA polymerases such as Polθ and Polλ were found to be required for the generation of Cas9-induced insertions [ 40, 42] . Whether such mechanisms are responsible for the generation of insertions in the *Ig* locus is still unknown. As insertion events are less frequent than deletion events during SHM [35], the elucidation of the mechanism of insertions could be a huge challenge.

The mechanism that inserts large fragments in the V region could be different from the mechanism that generates small insertions/duplications. A yeast mutant lacking Dna2 nuclease showed frequent insertions of large fragments (0.1–1.5 kb) in DSBs [43]. The inserted fragments are duplications, as they are not lost from their original loci. These insertions are dependent on NHEJ and Pol4 [43]. The mechanism involving Dna2 nuclease could probably explain the mechanism of large insertion observed in the anti-malaria antibodies [31]. The understanding of the mechanisms that contribute to the various types of rare indels would be useful to help increase the chances of eliciting and selecting bnAbs during infection or vaccination.

## Extraordinarily Long CDR H3 Antibodies Are Rare

The CDR H3 is the most diverse part of the variable region, as it consists of the V, D, and J segments [4]. During the process of V(D)J recombination, DNA end processing and addition of nucleotides by Tdt further increase the diversity of the CDR H3 [ 2, 5] . B cells with long CDR H3 in the BCRs are likely to be auto/polyreactive and are normally negatively selected [44]. The human CDR H3 chain typically consists of 8 to 16 amino acids [45]. However, CDR H3 of extraordinarily lengths (24 to 37 amino acids) had been found in some rare anti-viral bnAbs such as anti-HIV bnAbs [20]. Long CDR H3 antibodies are also common in B cell repertoires of patients with autoimmune diseases such as Systemic Lupus Erythematosus (SLE) [ 46– 48] . The mechanisms that generate long CDR H3 antibodies are partially understood and will be discussed below.

### The significance of long CDR H3 in bnAbs

Structural studies of how anti-viral antibodies and antigens interact have provided clues for the development of effective vaccines against HIV and influenza [49]. HIV bnAbs targeting the HIV envelope can be classified into several classes depending on the epitope specificity (CD4-binding site, VIV2-glycan, V3 glycan high mannose patch, the gp41 membrane proximal external region, and the gp120-gp41 bridging region) [50]. bnAbs targeting the V1V2-glycan, such as PG9, PG16, CH01-04, PGT141-145, CAP256-VRC26, and PDGM1400 in particular, have extraordinarily long CDR H3 [50]. For example, the bnAbs in the CAP256-VRC06 lineage have CDR H3 of 35 to 37 amino acids [51]. Long CDR H3 provides an unusual protruding, anionic, and often tyrosine sulphated, which allows penetration of the HIV-1 glycan shield [ 51– 53] . As bnAbs with long CDR H3 are generally less mutated compared to bnAbs that target the CD4 binding site (discussed below), the selection of bnAb precursors with long CDR H3 is considered to be a more feasible HIV vaccine design strategy [50].

The VRC01 bnAb lineage is an example of bnAb lineages targeting the CD4 binding site of the HIV env that acquires extremely high levels of SHM during antibody maturation [25]. Together with a short signature of 5 amino acids in the CDR3 of the light chain, these rare antibody diversification characteristics become a major challenge for bnAb induction in the HIV vaccine design strategy. In a recent investigation on the evolution of the VRC01 antibody lineage, Bonsignori *et al*. [54] found that the VRC08 clade of VRC01 lineage acquires broad neutralizing activities through a duplication that extends the CDR H3 length. The lengthening of CDR H3 of VRC08 can overcome the N276 glycan barrier without shortening the CDR L1 [54]. By reconstructing the VRC01 lineage bnAb maturation, they highlighted the significance of long CDR H3 in neutralization and provided a possible route in HIV-1 vaccine designs to elicit bnAbs targeting the CD4 binding site epitope [54].

### Possible mechanisms of long CDR H3 generation

Several reports have suggested that long CDR H3s in bnAbs are products associated with V(D)J recombination processes, such as usage of long D and/or J segments, fusion of D-D segments, V _H_ replacements, and long nucleotide additions by Tdt [ 55, 56] . Analysis of human peripheral blood antibodies shows that long CDR H3 antibodies use limited germline genes, for example, the D2 and D3 gene families that encode the longest D genes and the J _H_6 gene which encodes the longest J gene are preferentially enriched in antibodies with long CDR H3 [55]. The D-D fusion is a rare V(DD)J recombination event that happens at a frequency of approximately 1/800 naive B cells [55]. Although the contribution of D-D fusion to HIV-1 bnAb CDR H3 poses challenges to observation due to their unique sequence, some HIV bnAbs are thought to arise as the result of D-D fusion; for example, PGT145 (CDR H3 contains 30 amino acids) displays 12 bp in D4-17 sequence with three mismatches and 11 bp D5-24 sequence with two mismatches [ 56, 57] . VH replacement occurs when the V gene of a rearranged V(D)J exon is replaced by an upstream V segment through a second V(D)J recombination process [56]. The elongation of the CDR H3 structure is achieved when the short stretch from previously rearranged VH genes is left in the new CDR H3, leaving VH footprints that could thus be used to analyze VH replacement frequency [ 58, 59] . VH replacement occurs more frequently than D-D fusions with a 5.7% frequency in footprint analysis [60]. Many HIV-1-related bnAbs with long CDR H3 such as the PGT-class antibodies exhibit a considerable amount of VH footprints. Thus, it was reported that VH replacements contribute to long CDR H3 [56]. Together, these evidence, which shows that long CDR H3 antibodies are generated during V(D)J recombination, suggests that bnAbs with long CDR H3 can be selected in the naïve B cell repertoire.

While many lines of evidence point towards the V(D)J recombination process in generating long CDR H3, it is also possible that the process of SHM could contribute to long CDR H3. However, such evidence is lacking partly because of the heterogeneity of the CDR3 sequences in an antibody repertoire. In theory, SHM-initiated insertions discussed above could extend the CDR H3 length but the experiments to prove this theory would be challenging,as the frequency of SHM-initiated insertions are extremely low. Recent studies, showing that IgG antibodies (AID-exposed B cells) of anti-HIV bnAb producers have longer CDR H3s compared to healthy controls, may support the notion that long CDR H3 antibodies are generated during the SHM process [61]. Whether AID-initiated insertions can contribute to long CDR H3 in bnAbs still awaits further investigation. If SHM process could contribute to the generation of long CDR H3, activated B cell repertoire would be a target for selecting bnAbs in HIV vaccine design strategies.

## Improbable Mutations Are Rare Mutations onAID-poorly-favored DNA Sequence

The CDRs are the most hypermutated region in the *Ig* variable region, which is likely due to the availability of AID-intrinsically preferred DNA targets [ 35, 62] . It is known that certain codons of serine such as AGC, are better targets of AID than other codons of the same amino acid, AGT, TCT, TCC, TCA, and TCG [63]. Hence, the AGC codon is preferentially found in the CDRs, while the less mutable codons are found in the framework regions [63]. Rogozin and Kolchanov [64] reported that the underlined sequence in R GYW (R=A/G, Y=C/T, W=A/T) and T AA sequence motifs are mutational hotspots. In this context, RGYW is an AID-preferred DNA motif [ 65– 67] and mutation at the TAA motif is due to activities of error-prone DNA polymerase η [68]. Mutations at AID-less-preferred substrate are considered improbable mutations and many bnAbs such as the anti-HIV bnAbs targeting the CD4-binding site acquire improbable mutations [ 36, 69] . This might explain why bnAbs are rare and the elicitation of bnAbs remains a major obstacle to vaccine design.

### The significance of improbable mutations in bnAbs

Key improbable mutations are most likely the rate limiting step in the development of bnAbs [36]. Improbable mutations are identified by the intrinsic SHM patterns of the human germline V gene *passenger-Ig* mouse model system [36] or predicted based on the known AID-favored or poorly-favored DNA sequence [69]. In the study by Hwang *et al*. [36], the intrinsic mutation frequencies of a human germline VH1-2 were first determined through high throughput sequencing of GC B cells. By comparing the recurrent mutations in the VRC01-class bnAbs that use germline VH1-2 to the intrinsic mutation frequencies obtained from the *passenger-Ig*, improbable or rate-limiting mutations were determined. Wiehe *et al*. [69], on the other hand, determined improbable mutations using a computational program, ARMADiLLO, which is based on the probability of an amino acid substitution. They showed that reverting the improbable mutations in VRC01 antibodies to germline reduced their neutralization potency [69]. Since probable mutations are usually easier to obtain intrinsically, the rarer improbable mutations are considered the key bottlenecks, which determine the development of bnAb neutralization breadth [69]. The identification of improbable mutations provides insights into the role of DNA sequence in bnAb development. Vaccine strategies targeting the most rate-limiting step and the selection of a specific subset of improbable mutations could help accelerate the development of bnAbs.

A proof of concept that immunization strategies can be designed to select B cells carrying specific improbable mutations has recently been shown [70]. The anti-HIV1 bnAb DH270 lineage carries an improbable mutation, G57R, which occurs at an early stage of affinity maturation [71]. Reversion of this improbable mutation to the germline sequence abrogated neutralization activities, and G57R mutation alone was sufficient to confer reactivity against autologous virus and acquired heterologous neutralization breadth, indicating the importance of this improbable mutation [71]. Other mutations that occurred on AID-preferred hotspots in the DH270 lineage are dispensable for neutralization activities. Vaccination of humanized Ig mice with immunogens that can bind with moderate affinity to bnAb precursor lineage to activate it and with high affinity to B cell precursors that have acquired improbable mutations, could initiate selection of improbable mutations to improve antibody affinity maturation [70]. Together, these studies show that elicitation of bnAbs that carry crucial improbable mutations, which takes a long time (4.5 years) to expand, may be accelerated by specific immunogen design that targets the selection of improbable mutations. Thus, defining improbable mutations in a bnAb lineage is useful to vaccine design strategies.

### The enigma of DNA sequence mutability

Although it is known that certain motifs such as AGCT are more mutable than others, it is still not known why the same motif at different locations in a short sequence has different mutability. For example, the same AGCT motif in the CDRs has a higher mutation frequency than the same AGCT motif in the framework regions [35]. The same phenomenon was also observed in *non-Ig* passenger allele sequences [35]. These observations suggest that there are elements that can enhance or suppress SHM at AID-preferred motifs and that these elements are not specialized to the V exon sequence. Currently, it is still unknown what these elements are, which is a topic of active research. In this case, enhancer-like sequences, such as DIVAC and diversification activator, have been shown to be able to increase SHM of a flanking transcribed region by two orders of magnitude or more [ 72, 73] . Whether these DIVAC-mediated elements play a role in differential mutability of DNA sequence in the Ig variable exon remains elusive. Revealing the *cis*-elements that can enhance SHM may be useful to increase the mutation frequency of improbable mutations (discussed in the previous section).

In a related study, Senigl *et al*. [74] identified topological associated domains (TADs) in the genome that are more susceptible to SHM than others. These “hot” TADs have unique features such as enrichment for cohesion loader NIPBL, super-enhancers, and RNA polII pausing/stalling factors. Importantly, insertion of a strong Ig targeting element into a cold TAD makes it hot [74]. This study showed that SHM susceptibility may involve combinations of enhancer-like sequences and the genomic architecture [74].

## Perspectives

Here, we discuss a few types of rare mutational outcomes that can occur during the process of antibody diversification, including indels, long CDR H3s, and improbable mutations. These unconventional routes to alter antibody genes have implications in the generation and development of highly potent antibodies with broad neutralization breadths. As the study of these rare mutational events remains challenging, plenty of questions still remain to be answered, for example, the mechanisms underlying these mutational outcomes. Answering these questions and discovering the mechanisms behind rare routes of SHM impacting the generation of bnAbs will significantly influence the direction of vaccine design strategies, which is especially vital in the global pandemic world that we are living in today.
